# Diffusion tensor imaging reveals diffuse white matter injuries in locked-in syndrome patients

**DOI:** 10.1371/journal.pone.0213528

**Published:** 2019-04-10

**Authors:** Mylene Leonard, Felix Renard, Laura Harsan, Julien Pottecher, Marc Braun, Francis Schneider, Pierre Froehlig, Frederic Blanc, Daniel Roquet, Sophie Achard, Nicolas Meyer, Stephane Kremer

**Affiliations:** 1 Service d’imagerie 2, Hopitaux Universitaires de Strasbourg, Strabourg, France; 2 Faculté de medecine, Université de Strasbourg, Strasbourg, France; 3 Unité IRM 3T-Recherche-IRMaGE-Inserm US 17/CNRS UMS 3552, Université de Grenoble-Alpes, Grenoble, France; 4 Laboratoire MATICE-Pôle Recherche, CHU de Grenoble, Grenoble, France; 5 EA AGEIS, Univ. Grenoble-Alpes, Grenoble, France; 6 Engineering science, computer science and imaging laboratory (ICube), Integrative Multimodal Imaging in Healthcare, UMR 7357, University of Strasbourg-CNRS, Strasbourg, France; 7 Department of Biophysics and Nuclear Medicine, University Hospital Strasbourg, Strasbourg, France; 8 Service d’Anesthésie-Réanimation Chirurgicale, Hopitaux Universitaires de Strasbourg, Strasbourg, France; 9 Fédération de Médecine Translationnelle de Strasbourg (FMTS), Faculté de medecine, Université de Strasbourg, Strasbourg, France; 10 EA3072, Université de Strasbourg, Strasbourg, France; 11 Service de Neuroradiologie, CHRU de Nancy, Nancy, France; 12 Département d’anatomie, Faculté de medecine, Université de Lorraine, Nancy, France; 13 Inserm U947, Université de Lorraine, Nancy, France; 14 Service de Réanimation Médicale, Hopitaux Universitaires de Strasbourg, Strasbourg, France; 15 Inserm U1121, Université de Strasbourg, Strasbourg, France; 16 Service de neurochirurgie, Hopitaux universitaires de Strasbourg, Strasbourg, France; 17 Laboratoire ICube, Strasbourg, France; 18 Service de gériatrie, Hopitaux universitaires de Strasbourg, Strasbourg, France; 19 CNRS, Université de Grenoble Alpes, Grenoble, France; 20 GMRC, Service de Santé Publique, Hopitaux universitaires de Strasbourg, Strasbourg, France; University of Pécs Medical School, HUNGARY

## Abstract

Locked-in syndrome (LIS) is a state of quadriplegia and anarthria with preserved consciousness, which is generally triggered by a disruption of specific white matter fiber tracts, following a lesion in the ventral part of the pons. However, the impact of focal lesions on the whole brain white matter microstructure and structural connectivity pathways remains unknown. We used diffusion tensor magnetic resonance imaging (DT-MRI) and tract-based statistics to characterise the whole white matter tracts in seven consecutive LIS patients, with ventral pontine injuries but no significant supratentorial lesions detected with morphological MRI. The imaging was performed in the acute phase of the disease (26 ± 13 days after the accident). DT-MRI-derived metrics were used to quantitatively assess global white matter alterations. All diffusion coefficient Z-scores were decreased for almost all fiber tracts in all LIS patients, with diffuse white matter alterations in both infratentorial and supratentorial areas. A mixture model of two multidimensional Gaussian distributions was fitted to cluster the white matter fiber tracts studied in two groups: the least (group 1) and most injured white matter fiber tracts (group 2). The greatest injuries were revealed along pathways crossing the lesion responsible for the LIS: left and right medial lemniscus (98.4% and 97.9% probability of belonging to group 2, respectively), left and right superior cerebellar peduncles (69.3% and 45.7% probability) and left and right corticospinal tract (20.6% and 46.5% probability). This approach demonstrated globally compromised white matter tracts in the acute phase of LIS, potentially underlying cognitive deficits.

## Introduction

Locked-in syndrome (LIS) was defined by Plum and Posner in 1966 as a condition in which selective supramotor de-efferentiation produces paralysis of all four limbs and the lower cranial nerves without interfering with consciousness [1]. The patients are “locked” in their body, and their only possibility of communicating is to use vertical eye movements and blinking. LIS can be divided into three categories depending on the extent of motor impairment: classical LIS refers to Plum and Posner’s definition of patients with total immobility except for vertical eye movements and blinking; incomplete LIS refers to patients with preserved voluntary movements, other than vertical eye movements and blinking; total LIS refers to patients with total immobility including all eye movements [2].

LIS is usually secondary to a bilateral ventral pontine lesion or more rarely a mesencephalic lesion. The most frequent etiology is vascular, either ischaemia or haemorrhage [3]. Neuropathological studies of autopsied cases have helped to understand the pathophysiology of the syndrome [4,5]. Preserved consciousness depends on the sparing of specific cortical areas such as the frontoparietal network and the thalamocortical pathways [6,7]. Disruption of corticospinal and corticobulbar tracts for the nuclei of lower cranial nerves during their course through the ventral part of the pons induces quadriplegia and paralysis of the lower cranial nerves. Lateral eye movements are generally impossible due to damage to either the lateral gaze centre (adjacent to the nucleus of the abducens nerve) or its connexions with the cortex. However, vertical eye movements are spared because the vertical gaze centre is located in or just above the superior colliculi. Finally, LIS patients usually have partially preserved cutaneous sensation. The medial lemniscus located on the dorsal side of the pyramidal tracts is usually disrupted, but the more laterally located spinothalamic tract is generally spared.

Cognitive functions in LIS survivors have been explored by few neuropsychological studies. Very recent investigations [8–13] in LIS patients have highlighted moderate and selective cognitive impairments, some of which seem to be related to supratentorial cortical involvement.

The broad spectrum of magnetic resonance imaging (MRI) contrast modalities makes MRI one of the most powerful and flexible imaging tools for highlighting cerebral damage. Indeed, morphological MRI, based on conventional T_1_- or T_2_-weighted contrasts, is classically used for precise anatomical location of brainstem lesions, responsible for the LIS [14,15]. We explored the potential of diffusion tensor magnetic resonance imaging (DT-MRI) and a subsequent tract-based statistics approach applied to DT-MRI-derived metrics. This allowed us to quantitatively assess white matter integrity and to determine potential microstructural injuries. Probing the diffusion of water molecules in the tissues, DT-MRI and tensor-derived indices [16,17] offer a unique possibility to evaluate microstructural changes of the brain white matter. More importantly, these non-invasive data are contributing new insight into the structural network architecture of brain white matter in vivo. The diffusion process can be described by different coefficients. The two most common coefficients are fractional anisotropy (FA) and mean diffusivity (MD). FA measures the degree of anisotropy of a diffusion process and reflects the structural integrity within fiber tracts and may highlight axonal loss. MD measures the average diffusivity of water molecules. It is therefore influenced by cell size and integrity and highlights demyelination. These two indexes provide different but complementary information about water molecule diffusion motion and can provide details on the size, shape and geometry of brain tissues. Two other coefficients depicting the diffusion process are axial and radial diffusivity (RD). Axial diffusivity (AD) corresponds to the diffusivity in the main direction of the diffusion process. It was shown to be sensitive to axonal injury and loss. RD measures the mean diffusivity perpendicular to the main diffusion direction, and therefore informs on myelin sheath properties [18–21].

DT-MRI has already been applied in different pathological conditions to highlight abnormalities in white matter fiber tracts [22]. Surprisingly, LIS was not much investigated in DT-MRI studies: only two cases have been described in the literature, with a single patient in each report. One LIS patient was reported to have MD and FA abnormalities in the ventral pons, particularly in the corticospinal tract [23]. Another LIS patient demonstrated corticospinal tract integrity, specifically in the lateral areas of pontine infarcts, with the DT-MRI exam in a chronic LIS condition, associated with distal motor recovery [24].

Given the paucity of DT-MRI data in LIS patients, the primary objective of the present study was to provide a comprehensive and updated analysis of cerebral white matter injuries in a sample of seven consecutive LIS patients admitted to Strasbourg University Hospital from 2013 to 2016. To this end, we analysed whole brain white matter fiber tracts using DT-MRI in these locked-in patients, all of whom suffered from a lesion in the ventral part of the pons.

The secondary objective was to validate the DT-MRI results obtained with an anatomical analysis of white matter fiber tracts crossing the lesion on morphological MRI.

## Materials and methods

This study was approved by our Institutionnal Review Board (N° Comité de Protection des Personnes Est IV 08/53, N° Comité de Protection des Personnes Est IV: 08/54). It is registered on the “National agency of Security of Drug and the Health Products” database under the “2008-A00811-54” and “2008-A00694-51” EudraCT identifier.

This was a prospective single-center study, conducted between 2013 and 2016, at Strasbourg University Hospital, Strasbourg, France.

### Patients

After written informed consent was obtained from next of kin, seven patients (five males, two females; mean age, 50 years range 37–70 years) presenting a pontine lesion with LIS were included in the study.

The patients met the following inclusion criteria:

- They exhibited LIS clinically, as primarily assessed by an intensivist (JP) and subsequently confirmed by a referent rehabilitation physician of the awakening care unit of Strasbourg University Hospital (PF). LIS diagnosis criteria included a state of quadriplegia associated with lower cranial nerve paralysis. It also implied signs of consciousness through demonstration of clinical response to simple orders: opening eyes on request, and/or moving a part of the body on request and/or communicating with eye or head movements. The clinical characteristics of consecutive LIS patients are summarised in Table 1.
- They developed an anatomical lesion located in the ventral part of the pons without significant supratentorial lesions as assessed on morphological MRI sequences by an anatomist (MB) who analysed the injured anatomical structures.

**Table 1:** Clinical characteristics of LIS patients.

LIS PATIENTS	GENDER (male, female)	AGE (years)	ACCIDENT ETIOLOGY	DATE OF MRI/PERIOD BEFORE MRI (Days)	TIME TO LIS DIAGNOSIS (Days)	CLINICAL LIS DIAGNOSIS	CLINICAL EVOLUTION
**1**	F	70	Ischemic brainstem stroke on basilar artery thrombosis	24/04/13 D40	D1	LIS: Tetraplegia, lower cranial nerve paralysis Response to simple orders: Opening eyes on request, responses with blinking eyes	Death 24/07/13
**2**	M	47	Ischemic brainstem and cerebellar stroke on basilar artery thrombosis	10/04/13 D13	D6	INCOMPLETE LIS: Tetraplegia, lower cranial nerves paralysis. Response to simple orders: Opening eyes on request, left foot movements on stimulation	Death 24/07/13
**3**	M	57	Posterior fossa hemorrhage and secondary brainstem ischemic stroke	21/01/15 D16	D5	INCOMPLETE LIS: Tetraplegia, lower cranial nerves paralysis. Response to simple orders: Opening eyes on request, thumb movements on request, YES/NO response with head	Death 27/03/2015
**4**	M	37	Brainstem hemorrhage on unbalanced HTA	03/04/13 D22	D7	INCOMPLETE LIS: Tetraplegia, lower cranial nerves paralysis. Response to simple orders: Opening eyes on request, little upper and lower limb movements, YES/NO response with eyes	Steady state in LIS
**5**	M	39	Brainstem hemorrhage on unbalanced HTA	15/05/13 D41	D16	TOTAL LIS: Tetraplegia, lower cranial nerve paralysis. Response to simple orders: Opening eyes on request	Death 15/10/13
**6**	M	43	Brainstem hemorrhage on arterio-venous malformation	13/03/13 D10	D1	INCOMPLETE LIS: Tetraplegia, lower cranial nerves paralysis. Response to simple orders: Opening eyes on request, hangs of gaze, YES/NO response with eyes, left thumb movements	Walk with technical aids: tetraparesis
**7**	F	56	Ischemic brainstem on distal basilar artery thrombosis	10/05/16 D43	D6	INCOMPLETE LIS: Tetraplegia, lower cranial nerves paralysis Response to simple orders: YES/NO response with head, right upper limb movements on request	Steady state in LIS

All LIS patients suffered from an infratentorial lesion including the ventral part of the pons.

Six LIS patients did not have supratentorial lesions on morphological MRI images. Only one patient (LIS patient 6) had very mild supratentorial lesions concerning posterior hypothalamic and habenular nuclei. This patient was included in the study, however, because the supratentorial lesions were very localised and could not account for the extensive supratentorial white matter signal abnormalities on DT-MRI. The results are presented in Table 2.

**Table 2:** Cerebral anatomical injuries detected on morphological MRI sequences.

**LIS patients/ Lesions topography**	**1**	**2**	**3**
**Pons (left or right side of the pons)**	Corticospinal tract, Medial lemniscus, Parietotemporopontine tract, Ventral trigeminothalamic tract, Middle cerebellar peduncle, Frontopontine tract, Gigantocellular reticular nucleus, Pontine nuclei, B6 & B8 serotonergic cell groups, Medial longitudinal fasciculus, Corticopontine tract, Nucleus of abducens nerve, Trapezoid body, Central tegmental tract	Corticospinal tract, Medial lemniscus, Parietotemporopontine tract, Ventral trigeminothalamic tract, Middle cerebellar peduncle, Pontine nuclei, Trapezoid body, Spinothalamic tract, Central tegmental tract, Superior olivary complex, B6 & B8 serotonergic cell groups, Caudal pontine reticular nucleus, Gigantocellular reticular nucleus, A5, A6, A7 noradrenergic cell groups, Lateral tegmental field, Medial tegmental field, Pontine nuclei, Rubrospinal tract, Tectospinal tract, Medial longitudinal fasciculus	Corticospinal tract, Medial lemniscus, Parietotemporopontine tract, Ventral trigeminothalamic tract, Dorsal trigeminothalamic tract, Central tegmental tract, Spinothalamic tract, Trapezoid body, Locus Caeruleus (A6) + subcaerulean nucleus, Dorsal catecholaminergic tract, Longitudinal dorsal fasciculus, A5 & A7 noradrenergic cell groups
**Supratentorial region**	No injuries	No injuries	No injuries
**LIS patients/ Lesion topography**	**4**	**5**	
**Pons (left or right side of the pons)**	Corticospinal tract, Medial lemniscus, Parietotemporopontine tract, Ventral trigeminothalamic tract, Dorsal trigeminothalamic tract, Middle cerebellar peduncle, B3 & B4 serotoninergic cell groups, B6 & B7 serotoninergic cell groups, Medial longitudinal fasciculus, Nucleus of abducens nerve, Genu of facial nerve (VII), Rubrospinal tract, Central tegmental tract, Tectospinal tract, Locus Caeruleus (A6) + subcaerulean nucleus, Mesencephalic tract of trigeminal nerve	Corticospinal tract, Medial lemniscus, Parietotemporopontine tract, Ventral trigeminothalamic tract, Dorsal trigeminothalamic tract, Middle cerebellar peduncle, Pontine nuclei, Trapezoid body, Spinothalamic tract, Central tegmental tract, Superior olivary complex, Nucleus of abducens nerve, Genu of facial nerve (VII), B3 & B4 serotoninergic cell groups, B6 & B7 serotoninergic cell groups, Medial longitudinal fasciculus, Locus Caeruleus (A6) + subcaerulean nucleus, Medial superior central nucleus, Lateral superior central nucleus, Mesencephalic nucleus and tract of trigeminal nerve	
**Supra tentorial region**	No injuries	No injuries	
**LIS patients/ Lesions topography**	**6**	**7**	
**Pons (left or right side of the pons)**	Medial lemniscus, Ventral trigeminothalamic tract, Dorsal trigeminothalamic tract, Locus Caeruleus (A6) + subcaerulean nucleus, Mesencephalic nucleus and tract of trigeminal nerve, Superior cerebellar peduncle, Medial longitudinal fasciculus, Central tegmental tract, Medial superior central nucleus, Lateral superior central nucleus, Spinothalamic tract, Ventral nucleus of the lateral lemniscus, Lateral lemniscus	Medial lemniscus, Spinothalamic tract, A8 dopaminergic cell group Pontine nuclei, Vestibular nucleus, Collicular nucleus, Pontine reticular nucleus, Tectospinal tract, Periaqueductal grey substance, Medial longitudinal fasciculus, Frontopontin tract	
**Left side of the midbrain**	Periaqueductal grey substance, Inferior colliculus, Spinothalamic tract, Ventral trigeminothalamic tract, Dorsal trigeminothalamic tract, Central tegmental tract, Lateral mesencéphalic nucleus, B3 & B4 serotoninergic cell groups, B6 & B7 serotoninergic cell groups, A8 noradrenergic cell groups, Tegmental olivary tract, Medial lemniscus, Superior colliculus, Medial geniculate body	No injuries	
**Supratentorial region**	Posterior hypothalamic nucleus Habenular nuclei	No injuries	

#### Healthy volunteers

Nineteen healthy volunteers (thirteen males, six females; mean age, 31 years range 19–51 years) were included in the study after written informed consent was obtained.

### Data acquisition

All LIS patients and healthy volunteers underwent a brain MRI examination, which was performed using a 1.5-Tesla scanner (Avanto, Siemens, Erlangen, Germany) and an eight-channel head coil. The LIS patients were scanned a few days (26 ± 13 days) after the causative brain injury, when sedative drug withdrawal allowed for spontaneous ventilation.

#### LIS patients

- DTI acquisition: 30 gradient diffusion directions, b = 1000 sec/mm^2^, TR = 6800 ms, TE = 99 ms, FOV = 230×230 mm^2^, matrix 128×128, 41 slices, slice thickness: 3.5 mm.
- Morphological analyses:
  - Diffusion: 12 directions, b = 1000 sec/mm^2^, TR = 4500 ms, TE = 96 ms, matrix 128×128, 30 slices, slice thickness: 3 mm.
  - T1 3D MPRAGE: TR = 1900 ms, TE = 2.68 ms, TI = 1100 ms, angle = 15°, matrix 320×320, 160 slices, slice thickness: 1 mm.
  - Axial FLAIR: TR = 9000 ms, TE = 126 ms, TI = 2500 ms, matrix 384×384, 25 slices, slice thickness: 4 mm.
  - Axial gradient echo T2: TR = 1120 ms, TE = 15 ms, angle = 20°, matrix 256×256, 25 slices, slice thickness: 4 mm.
  - Axial T2 thin posterior fossa: TR = 5030 ms, TE = 117 ms, matrix 320×320, 25 slices, slice thickness: 3 mm.

#### Healthy volunteers

- DTI acquisition: 30 gradient diffusion directions, b = 1000 sec/mm^2^, TR = 6800ms, TE = 99 ms, FOV = 230×230 mm^2^, matrix 128×128, 41 slices, slice thickness: 3.5 mm
- T1 3D MPRAGE: TR = 1900 ms, TE = 2.68 ms, TI = 1100 ms, angle = 15°, matrix 320×320, 160 slices, slice thickness: 1 mm.

### DTI analyses

Data pre-processing: Diffusion MRI data were corrected for eddy currents and motion artefacts, then skull-stripped using FSL software [25]. DT-MRI metrics, including FA and different diffusion coefficients (MD, AD, RD) were further estimated [26].

In the next step, each FA map was registered on a common template (FMRIB58_FA provided by FSL.) Other scalar images were then co-registered using FA map transformation.

For each white matter anatomical region provided by the JHU DTI-based white-matter atlases [27] (48 white matter fiber tracts) previously transformed on the common template, the mean of each coefficient was estimated. To consider only white matter and to avoid partial volume with grey matter, only voxels inside the skeleton of white matter were included in the average estimation. For this purpose, the tract-based spatial statistics method was used to estimate the white matter skeleton [28].

**Fig 1:**
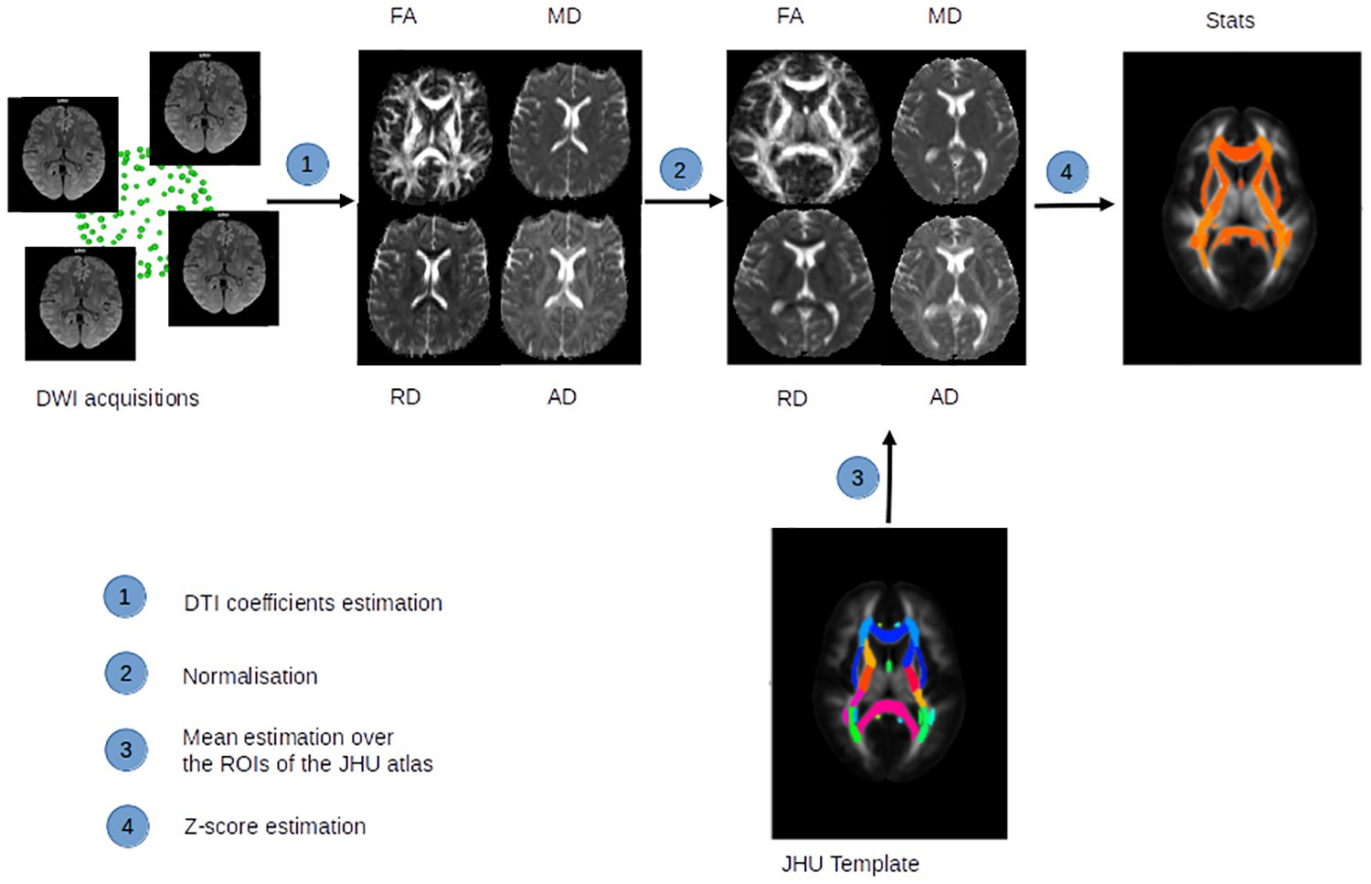
Presentation of the framework of the analysis. Diffusion weighted images were acquired, then: 1) Four DT-MRI coefficients (FA, MD, RD and AD) were estimated in the patient space. 2) The transformation between the patient’s images and the atlas was calculated on the FA map and then applied to the different coefficient maps. 3) The mean of the patient diffusivity coefficients was estimated over the different regions of interest of the JHU atlas, 48 white matter fiber tracts. 4) Finally Z-score statistics were calculated for all ROIs of the JHU atlas and for the different DT-MRI coefficients.

Finally, Z-scores were calculated for the LIS patients for each DT-MRI index and for each region of interest (ROI) of the template (Fig 1). Since the Z-score is a normalised measure, a Z-score lower than −1.96 or higher than 1.96 can be considered as indicating a difference between the subject and the control.

### Statistical analyses

The data were analysed using Bayesian methods. In Bayesian statistics, a prior distribution is specified for the parameter to estimate. The data are used to update this distribution to obtain a posterior distribution that describes all that is known about the parameter. Bayesian methods also make it possible to work on very small data sets by partially compensating for the lack of information provided by the small sample size using prior information. Moreover, Bayesian methods can easily provide probabilistic statements about the parameter of interest.

The model was built as follows: because of the limited number of subjects and the relatively large number of fiber tracts, the 48 white matter fiber tracts were considered as the statistical units whose data were measured simultaneously on FA and MD Z-score data and repeated across subjects. We hypothesised that it was possible to find a specific pattern of white matter fiber tract lesions for LIS patients. We therefore used a model that allows one to position the white matter fiber tracts in relation to each other in terms of the intensity of white matter lesions on all beams, according to both the FA and MD Z-score dimensions. This position is the parameter of interest in our Bayesian model. We then made the additional assumption that the nerve bundles would separate into two distinct groups. Group 2 represented the most injured white matter fiber tracts, whereas group 1 represented the least injured white matter fiber tracts. Thus, a given white matter fiber tract had a probability *p* of being in the group 1 and a probability *1*−*p* of being in group 2. White matter fiber tracts were classified as belonging to group 2 when the probability *1*−*p* was greater than 0.5, and belonging to group 1 otherwise. Statistical analyses were applied on FA and MD Z-scores data to highlight the most injured white matter fiber tracts. A multidimensional mixture of two Gaussian distributions was used to model the data.

The model likelihood is:

$$f(x)=p\left[\frac{1}{{\left(2\pi \right)}^{N/2}\Sigma^{1/2}}e^{-\frac{1}{2}{\left(x-\Gamma_{1}\right)}^{Τ}\Sigma^{-1}(x-\Gamma_{1})}\right]+(1-p)\left[\frac{1}{{\left(2\pi \right)}^{N/2}\Sigma^{1/2}}e^{-\frac{1}{2}{\left(x-\Gamma_{2}\right)}^{Τ}\Sigma^{-1}(x-\Gamma_{2})}\right]$$

with:

$$\{\begin{array}{ll} \Gamma_{1}= & \mu_{1}+{Α}_{1}\\ \Gamma_{2}= & \mu_{2}+{Α}_{2} \end{array}$$

where $\Gamma_{1},\Gamma_{2},\mu_{1},\mu_{2}\in {\mathbb{R}}^{2}$ and A_1_, A_2_ are two-dimensional vectors of two independent random Gaussian variables.

To take into account across-subject repetition, a random effect was added to the nerve bundle to model intra-bundle variability in addition to inter-bundle variability. Variability is thus considered on each of the two coordinates (FA and MD) of the mixture. Intra-bundle variability was assumed to be identical in the two components of the mixture. Intra-bundle variability was also constrained to be drawn from the same distribution on each component, i.e.:

$$\begin{array}{llll} {Α}_{1}=\left(\begin{array}{c} {Α}_{11}\\ {Α}_{12} \end{array}\right),{Α}_{2}=\left(\begin{array}{c} {Α}_{21}\\ {Α}_{22} \end{array}\right), & {Α}_{11},{Α}_{21}\;N(0,\sigma_{1}) & \text{and} & {Α}_{12},{Α}_{22}\;N(0,\sigma_{2}) \end{array}$$

#### Prior distributions

We used a low-information prior for *p*, with *p~Beta (1*,*2)*. The priors of the random effects variances were (100,100). The means *μ*_1_, *μ*_2_ were given Gaussian prior N(0.1000).

Finally, the prior of the inverse of the variance covariance matrix was a Wishart distribution with parameters 0.0001 (which corresponds to a variance of 1000) and 0 and it was considered identical in the two components of the mixture.

$$\Sigma =\left[\begin{array}{cc} 0,0001 & 0\\ 0 & 0,0001 \end{array}\right]$$

A sensitivity analysis on variance covariance matrix parameters was done to evaluate their weight on the final fiber tract classification.

The model was fitted using the Markov Chain Monte Carlo (MCMC) method and parameters were monitored through the MCMC samples. Computations were done with R 3.0.1. and WinBUGS 1.4.3 [29].

### Anatomical analyses

An anatomist (MB) analysed morphological MRI images, based essentially on FLAIR, T2 thin posterior fossa and diffusion-weighted images, and described white matter fiber tracts crossing the lesion (Table 2). The injured white matter fiber tracts were not visualised on morphological MRI images, but the knowledge of both their anatomical location and path made their identification possible.

We compared injured white matter fiber tracts highlighted by anatomical analysis and by diffusion tensor imaging to assess the correlations between these two MRI modalities. Doing so, we aimed to check whether injured pathways revealed by DT-MRI were also revealed by anatomical analyses on morphological MRI images.

## Results

### DTI analyses

**Fig 2:**
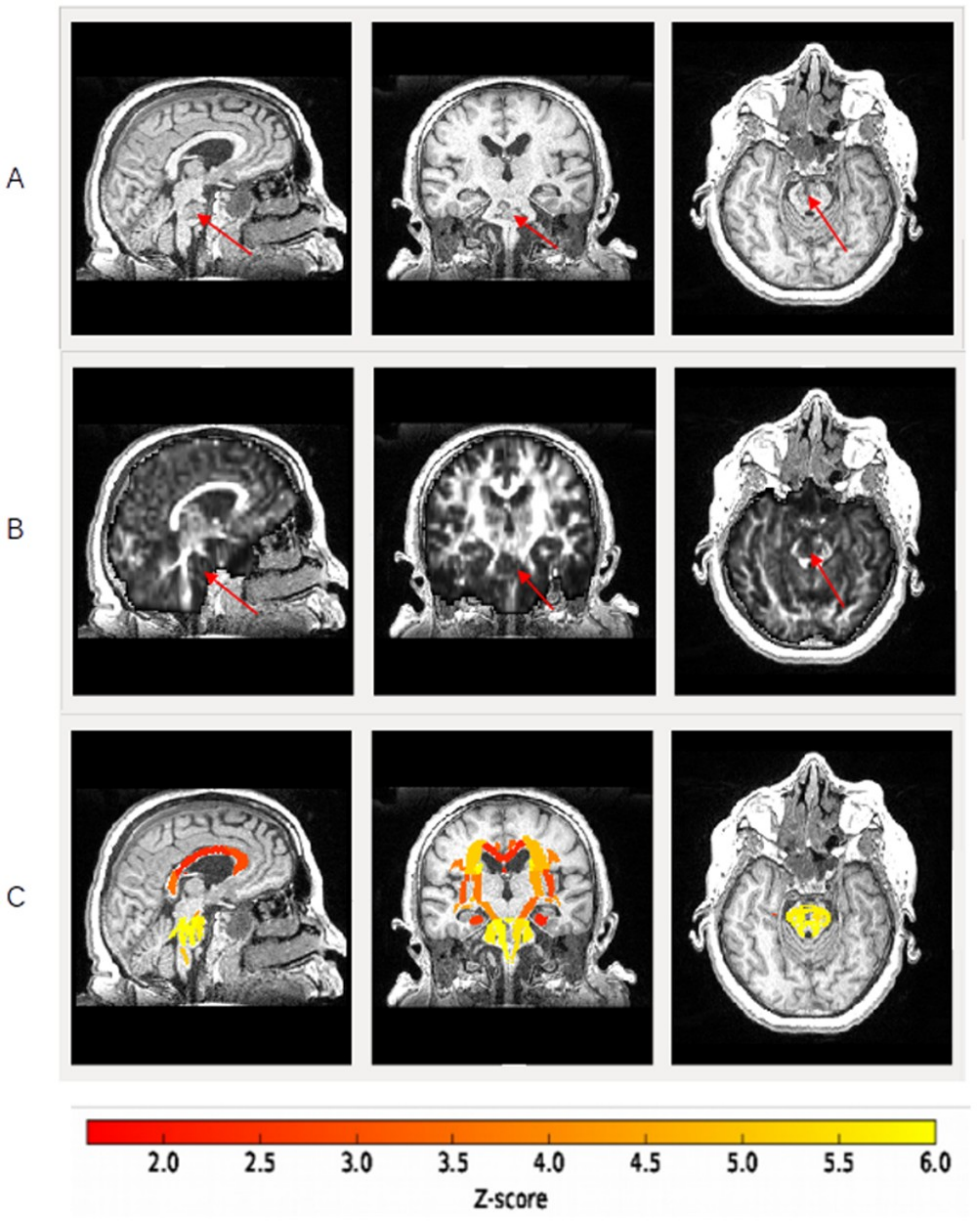
Z-score results. A: T1-weighted images of a LIS patient (sagittal, coronal and axial slices). Arrows indicate the lesion in the pons. B: FA images (sagittal, coronal and axial slices) of a LIS patient. Arrows indicate the lesion in the pons. C: Example of FA Z-score statistic (absolute values) on T1-weighted images (sagittal, coronal and axial slices) for a LIS patient and for the 48 white matter fiber tracts studied: yellow indicates the highest Z-scores and red the lowest Z-scores (see colour bar just under the image). The patient shows a severe injury at the pons level.

The Z-scores of all DT-MRI-derived parameters–FA, MD, RD and AD–were lower for almost all the white matter fiber tracts studied and for all LIS patients compared with control subjects. For instance, the diffusivity coefficient Z-score data are provided for the patient 6 in Tables 3 and 4. To summarise: the FA Z-scores were lower for the 48 nerve bundles, ranging from –2.33 to –10.75 (Fig 2). The MD Z-scores were lower for the 48 nerve bundles, ranging from –1.51 to –14.28. The AD Z-scores were lower for the 48 nerves bundles, ranging from –2.51 to –14.97. The RD Z-scores were overall lower for all the fiber tracts studied, ranging from 0.02 to –9.71.

**Table 3:** FA and MD Z-scores of the 48 white matter fiber tracts studied with DT-MRI for patient 6.

	FA		MD
**Superior fronto-occipital fasciculus R**	-10.7480	**Superior fronto-occipital fasciculus R**	-14.2809
**Superior fronto-occipital fasciculus L**	-9.9689	**Superior fronto-occipital fasciculus L**	-12.5544
**Medial lemniscus R**	-9.8877	**External capsule L**	-10.9676
**Superior cerebellar peduncle L**	-8.9701	**Superior corona radiata R**	-10.7517
**Medial lemniscus L**	-8.8790	**External capsule R**	-10.6353
**Corticospinal tract L**	-8.7662	**Superior corona radiata L**	-10.5439
**Fornix stria terminalis R**	-8.0366	**Cingulum cingulate gyrus L**	-9.9685
**Fornix stria terminalis L**	-8.0298	**Cingulum cingulate gyrus R**	-9.6954
**Corticospinal tract R**	-7.7230	**Uncinate fasciculus L**	-9.4649
**Superior corona radiata L**	-7.6792	**Superior longitudinal fasciculus L**	-9.2725
**Retrolenticular part of internal capsule L**	-7.0747	**Fornix Stria terminalis R**	-9.1615
**External capsule R**	-7.0704	**Pontine crossing tract**	-8.9053
**Superior cerebellar peduncle R**	-6.9633	**Anterior corona radiata R**	-8.8317
**External capsule L**	-6.8178	**Retrolenticular part of internal capsule L**	-8.7158
**Superior corona radiata R**	-6.8129	**Corticospinal tract L**	-8.5642
**Pontine crossing tract**	-6.6466	**Corticospinal tract R**	-8.4044
**Posterior limb of internal capsule R**	-6.5597	**Uncinate fasciculus R**	-8.1407
**Retrolenticular part of internal capsule R**	-6.5514	**Anterior corona radiata L**	-8.1271
**Tapetum R**	-6.4361	**Posterior corona radiata L**	-8.0524
**Posterior corona radiata R**	-6.4174	**Superior longitudinal fasciculus R**	-7.9398
**Posterior limb of internal capsule L**	-6.4132	**Medial lemniscus R**	-7.8712
**Posterior thalamic radiation L**	-6.2763	**Fornix Stria terminalis L**	-7.8311
**Cingulum cingulate gyrus L**	-6.0431	**Posterior corona radiata R**	-7.8086
**Splenium of corpus callosum**	-5.9731	**Retrolenticular part of internal capsule R**	-7.7027
**Cingulum cingulate gyrus R**	-5.9627	**Posterior thalamic radiation L**	-7.1591
**Posterior corona radiata L**	-5.9540	**Superior cerebellar peduncle L**	-6.6437
**Uncinate fasciculus L**	-5.8443	**Inferior cerebellar peduncle R**	-6.6215
**Superior longitudinal fasciculus L**	-5.7910	**Posterior limb of internal capsule R**	-6.5569
**Uncinate fasciculus R**	-5.6202	**Sagittal stratum L**	-6.1629
**Posterior thalamic radiation R**	-5.4262	**Posterior thalamic radiation R**	-6.0225
**Body of corpus callosum**	-5.2457	**Medial lemniscus L**	-5.7711
**Inferior cerebellar peduncle R**	-5.0904	**Anterior limb of internal capsule R**	-5.5304
**Genu of corpus callosum**	-4.9810	**Body of corpus callosum**	-5.2061
**Anterior corona radiata L**	-4.7811	**Posterior limb of internal capsule L**	-5.1651
**Tapetum L**	-4.6967	**Anterior limb of internal capsule L**	-4.9074
**Superior longitudinal fasciculus R**	-4.6390	**Cingulum hippocampus R**	-4.6596
**Anterior corona radiata R**	-4.5589	**Sagittal stratum R**	-4.4550
**Sagittal stratum L**	-4.4256	**Cingulum hippocampus L**	-4.2916
**Anterior limb of internal capsule R**	-4.3299	**Superior cerebellar peduncle R**	-4.1469
**Fornix column and body**	-4.1631	**Middle cerebellar peduncle**	-4.0506
**Sagittal stratum R**	-3.8284	**Inferior cerebellar peduncle L**	-3.9592
**Inferior cerebellar peduncle L**	-3.7371	**Tapetum L**	-3.4286
**Anterior limb of internal capsule L**	-3.6725	**Tapetum R**	-3.3919
**Cerebral peduncle L**	-3.5772	**Genu of corpus callosum**	-2.5437
**Middle cerebellar peduncle**	-3.4018	**Cerebral peduncle L**	-2.2949
**Cerebral peduncle R**	-3.3242	**Splenium of corpus callosum**	-2.0907
**Cingulum hippocampus R**	-2.6142	**Cerebral peduncle R**	-2.0068
**Cingulum hippocampus L**	-2.3326	**Fornix column and body**	-1.5109

The 48 white matter fiber tracts studied by DT-MRI are ranked from largest to smallest Z scores.

Abbreviations: R, right; L, left.

**Table 4:** AD and RD Z-scores of the 48 white matter fiber tracts studied with DT-MRI for patient 6.

	AD		RD
**Superior fronto-occipital fasciculus R**	-14.9662	**Superior fronto-occipital fasciculus R**	-9.7102
**Superior fronto-occipital fasciculus L**	-12.9125	**Superior fronto-occipital fasciculus L**	-9.0290
**External capsule L**	-10.8314	**Superior corona radiata R**	-7.7300
**External capsule R**	-10.3743	**Superior corona radiata L**	-7.3002
**Medial lemniscus R**	-10.0378	**External capsule L**	-7.2717
**Uncinate fasciculus L**	-9.6659	**Fornix Stria terminalis R**	-6.9321
**Superior cerebellar peduncle L**	-9.4621	**External capsule R**	-6.8906
**Uncinate fasciculus R**	-9.2492	**Cingulum cingulate gyrus R**	-5.8706
**Superior corona radiata L**	-9.2313	**Superior longitudinal fasciculus L**	-5.8536
**Superior corona radiata R**	-9.0758	**Pontine crossing tract**	-5.5758
**Pontine crossing tract**	-9.0168	**Retrolenticular part of internal capsule L**	-5.5576
**Medial lemniscus L**	-8.9807	**Posterior corona radiata L**	-5.5202
**Cingulum cingulate gyrus R**	-8.8075	**Cingulum cingulate gyrus L**	-5.4554
**Cingulum cingulate gyrus L**	-8.6000	**Anterior corona radiata R**	-5.4455
**Fornix stria terminalis R**	-8.4146	**Corticospinal tract R**	-5.4109
**Corticospinal tract L**	-8.3372	**Fornix stria terminalis L**	-5.3612
**Anterior corona radiata L**	-8.2151	**Anterior corona radiata L**	-5.2371
**Superior longitudinal fasciculus L**	-8.2051	**Retrolenticular part of internal capsule R**	-5.2280
**Anterior corona radiata R**	-7.8856	**Superior longitudinal fasciculus R**	-5.0616
**Retrolenticular part of internal capsule L**	-7.6847	**Corticospinal tract L**	-5.0024
**Posterior thalamic radiation L**	-7.6127	**Posterior corona radiata R**	-4.8591
**Fornix Stria terminalis L**	-7.6017	**Uncinate fasciculus L**	-4.8008
**Posterior corona radiata R**	-7.4006	**Uncinate fasciculus R**	-4.4650
**Posterior corona radiata L**	-7.3467	**Posterior limb of internal capsule R**	-4.0476
**Corticospinal tract R**	-7.1084	**Sagittal stratum L**	-3.8201
**Inferior cerebellar peduncle R**	-6.7659	**Posterior thalamic radiation L**	-3.7201
**Superior cerebellar peduncle R**	-6.7603	**Inferior cerebellar peduncle R**	-3.7108
**Superior longitudinal fasciculus R**	-6.6292	**Anterior limb of internal capsule R**	-3.6174
**Retrolenticular part of internal capsule R**	-6.6092	**Anterior limb of internal capsule L**	-3.3819
**Body of corpus callosum**	-6.3880	**Posterior limb of internal capsule L**	-3.2335
**Posterior thalamic radiation R**	-6.3447	**Medial lemniscus R**	-3.0974
**Posterior limb of internal capsule R**	-6.1456	**Body of corpus callosum**	-2.8863
**Anterior limb of internal capsule R**	-5.6379	**Superior cerebellar peduncle L**	-2.8499
**Sagittal stratum L**	-5.4333	**Middle cerebellar peduncle**	-2.7544
**Posterior limb of internal capsule L**	-5.0180	**Cingulum hippocampus L**	-2.6500
**Anterior limb of internal capsule L**	-4.9110	**Cingulum hippocampus R**	-2.5982
**Tapetum R**	-4.7050	**Posterior thalamic radiation R**	-2.4691
**Genu of corpus callosum**	-4.3934	**Tapetum L**	-2.1785
**Inferior cerebellar peduncle L**	-4.2853	**Sagittal stratum R**	-2.1423
**Tapetum L**	-4.1642	**Inferior cerebellar peduncle L**	-1.7199
**Sagittal stratum R**	-4.1065	**Tapetum R**	-1.6238
**Cingulum hippocampus R**	-4.0533	**Superior cerebellar peduncle R**	-1.3358
**Middle cerebellar peduncle**	-4.0163	**Cerebral peduncle L**	-1.1616
**Cingulum hippocampus L**	-3.9027	**Cerebral peduncle R**	-1.0224
**Fornix column and body**	-3.5530	**Medial lemniscus L**	-0.8470
**Splenium of corpus callosum**	-3.3389	**Fornix column and body**	-0.5755
**Cerebral peduncle L**	-2.7799	**Genu of corpus callosum**	-0.2206
**Cerebral peduncle R**	-2.5099	**Splenium of corpus callosum**	0.0200

The 48 white matter fiber tracts studied by DT MRI are ranked from largest to smallest Z-scores.

Abbreviations: R, right; L: left.

These Z-score data could be generalized for the other six LIS patients, given that the results were for the most part similar. In rare cases, white matter fiber tract Z-scores were not abnormal or were higher than in controls (S1–S3 Files). The data of the six other LIS patients are available from the Dryad Digital Repository: https://doi.org/10.5061/dryad.6j2c875.

**Fig 3:**
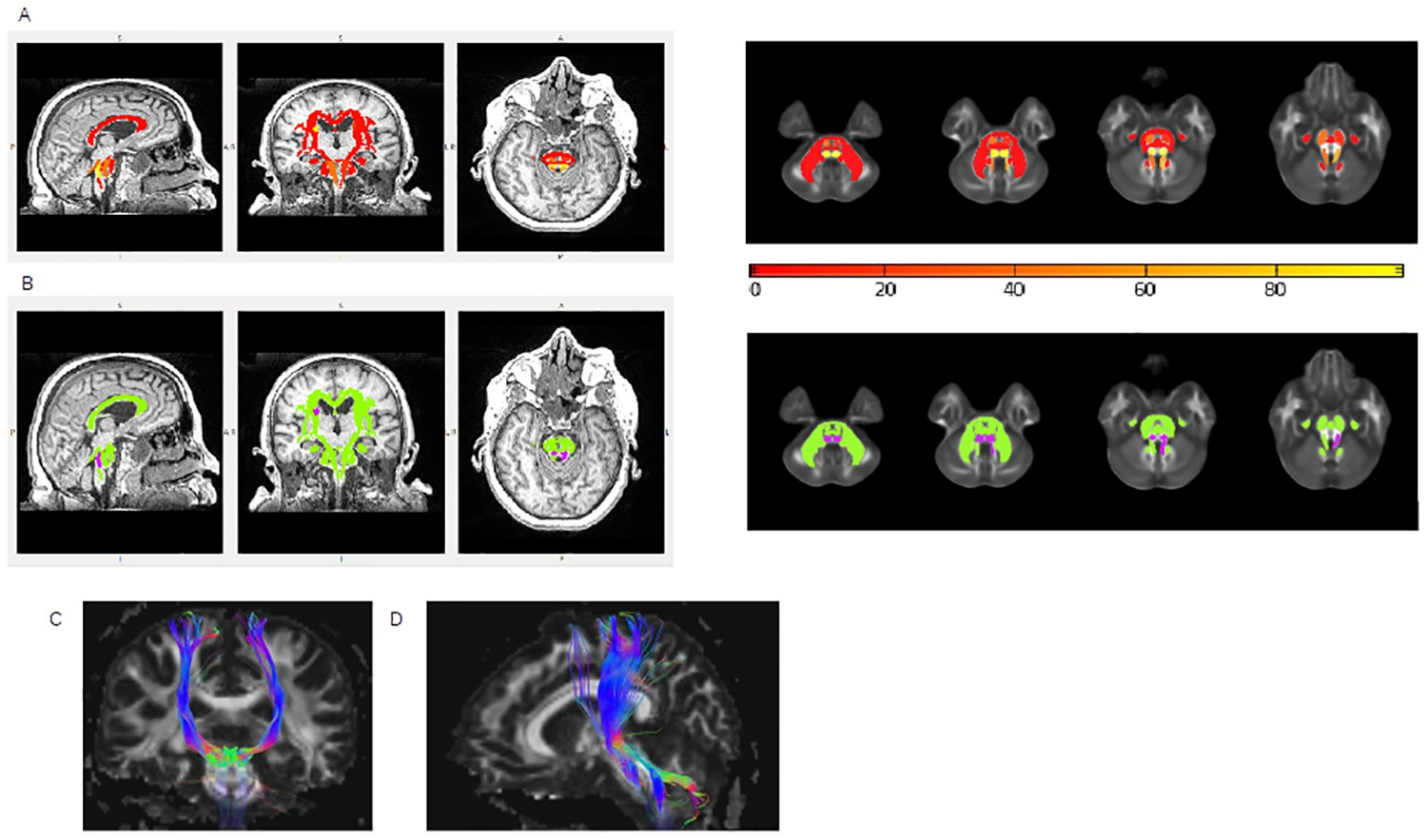
Separation of the 48 white matter fiber tracts into two groups. A: Probability map (sagittal, coronal, and axial T1-weighted slices on the brain and axial FA slices in the atlas space at the level of the pons) of belonging to the two different groups for the 48 white matter fiber tracts studied at the level of the population: yellow indicates the highest probability (>80%) of belonging to group 2 (the most injured white matter fiber tracts) and red the lowest probability (<20%) of belonging to group 2. B: Binary affiliation map (sagittal, coronal and axial T1-weighted slices on the brain and axial FA slices in the atlas space at the level of the pons) of the 48 white matter fiber tracts: in green those belonging to group 1, in purple those belonging to group 2, which are medial lemniscus and the left superior cerebellar peduncle. C: Tractography of the white matter fiber tracts that had the highest probability of belonging to group 2: corticospinal tracts (a), medial lemniscus and superior cerebellar peduncles (b).

The 48 nerve bundles studied were separated into two groups (group 2 representing the most injured white matter fiber tracts and group 1 representing the least injured white matter fiber tracts) according to both FA and MD Z-score data (S1 Table)(Fig 3).

For eight of the 48 white matter fiber tracts, the probabilities of belonging to group 2 were high and distributed as follows: left and right medial lemnisci had a 98,4% and 97.9% chance, respectively, of belonging to group 2, while the right superior frontooccipital fasciculus had a 99.5% and the left superior cerebellar peduncle had a 69.3% chance of belonging to group 2.

**Fig 4:**
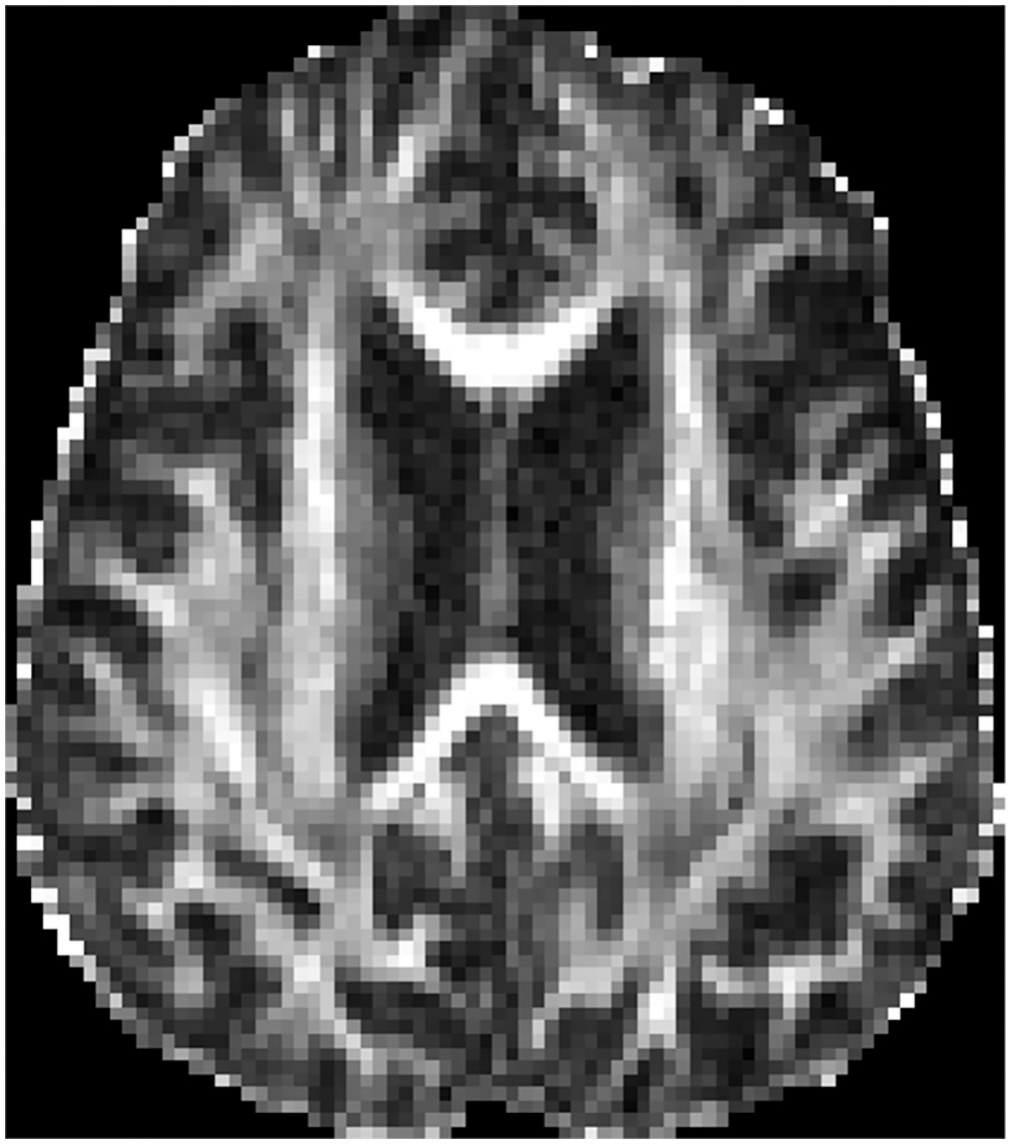
Representation of lateral ventricle atrophy in one of the LIS patients studied on an FA map image.

**Fig 5:**
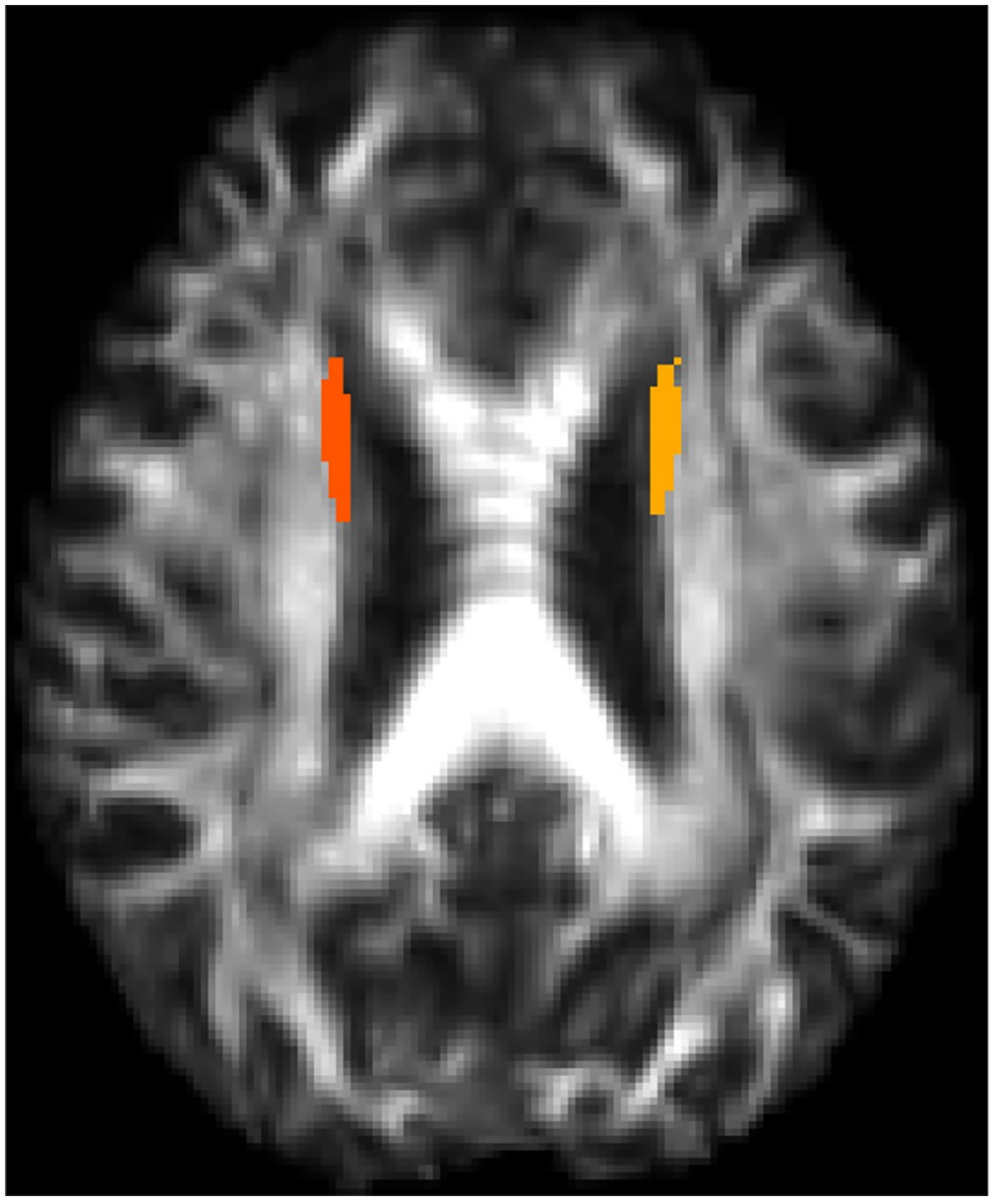
Colour marks represent ROI location for left and right superior frontooccipital tract analysis on an FA map image.

Four other fiber tracts also had a higher probability of belonging to group 2 than the others, although at a lower level than the first four, namely: right and left corticospinal tracts (46.5% and 20.6%, repectively), right superior cerebellar peduncle (45.7%) and the left superior frontooccipital fasciculus (26.5%). The superior frontooccipital fasciculi were erroneously categorised into the most injured white matter fiber tracts, because there was severe atrophy of the lateral ventricles, which caused misalignment of the superior frontooccipital tracts during DTI analysis. These artefacts are illustrated in Fig 4 where atrophy can be observed in the patient space and in Fig 5 after registration in the atlas space, where the ROIs concerned lay on the ventricles.

Conversely, the other 40 fiber tracts had very low probability (less than 10%) of belonging to Group 2.

### Anatomical analysis

**Fig 6:**
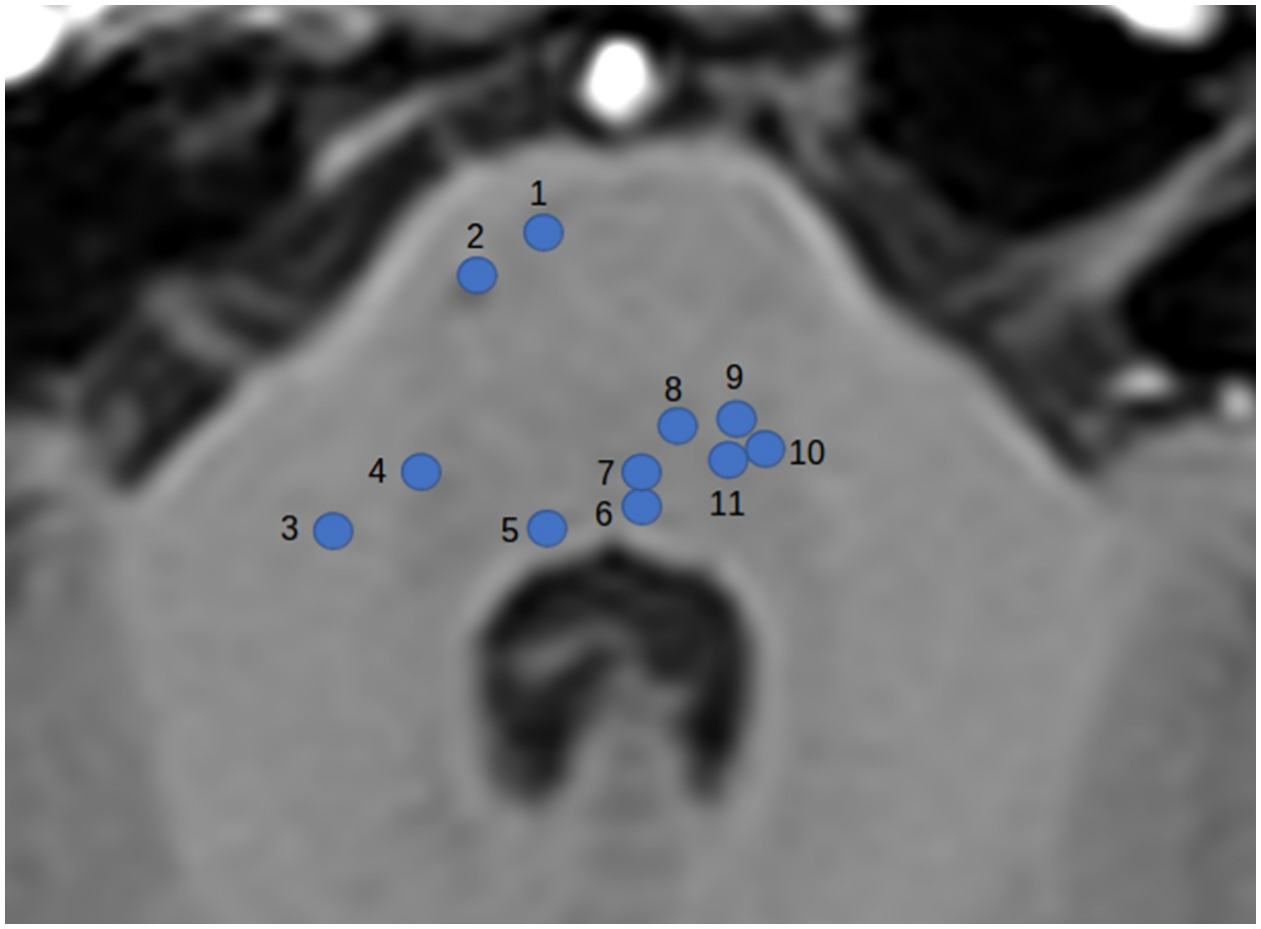
Axial T1 MRI image of the pons with location representation of some of the injured white matter fiber tracts detected by anatomical analysis. 1: Frontopontine tract; 2: Corticospinal tract; 3: Middle cerebellar peduncle; 4: Nucleus of trigeminal nerve; 5: Nucleus of abducens nerve; 6: Medial longitudinal fasciculus; 7: Tectospinal tract; 8: Medial lemniscus; 9: Lateral lemniscus; 10: Rubrospinal tract; 11: Central tegmental tract.

Medial lemniscus tract injuries, at the level of the pons were observed for all LIS patients (Table 2). Six LIS patients revealed ventral trigeminothalamic tract injuries and four had dorsal trigeminothalamic tract injuries. Five LIS patients exhibited corticospinal tract and parietotemporopontine tract injuries. Structural MRI images are available in supplementary files (S1–S7 Figs) for each LIS patient. Fig 6 illustrates the location of some of the injured white matter fiber tracts detected on morphological MRI images.

## Discussion

This study was designed to assess white matter fiber tract integrity using DT-MRI, both at the infratentorial and the supratentorial levels in LIS patients, unravelling the consequences of LIS-specific brain lesions on overall white matter integrity. We showed diffuse compromised supra- and infratentorial white matter fiber tracts in LIS patients. Moreover, we identified three pathways crossing the lesion responsible for LIS in the ventral part of the pons, which showed more injury than the other fiber tracts: the corticospinal tract, the medial lemniscus tract and the superior cerebellar peduncles.

Tract-based spatial statistics identified abnormal DT-MRI-derived parameter values, abnormal along tracts, indicating globally lower FA, MD, AD and RD Z-scores than in controls and suggesting highly compromised white matter integrity. FA describes the degree of directionality of water in the tissue, reflects structural integrity of white matter fiber tracts and is correlated with effective connectivity [30]. Therefore, lower FA hints at white matter fiber tract disorganisation. In the study, all LIS patients had lower FA Z-scores than control subjects for almost all white matter fiber tracts. The MRI results suggest that LIS patients suffered from global white matter fiber tract disorganisation. AD and RD provide additional information on white matter structures that is more specific to the underlying microstructural processes, as compared to FA. AD reflects parallel diffusivity along the axon and is altered in case of axonal injury [31]. In the present study, AD Z-scores were overall lower, showing global axonal injury. RD reflects diffusivity perpendicular to the axon and higher values were previously associated with demyelination processes of white matter tracts in humans [32] and in animal models [33,34]. The MD coefficient varies according to the progressive stage of the injury, giving an overall measurement of water molecule movements. In the acute phase, MD is decreased by intracellular oedema, whereas in the chronic phase MD is increased by leukoencephalopathy, combining demyelination, axonal loss and focal regions of necrosis [19,31]. Consequently, RD will increase with demyelination and MD will increase with the rise of water molecule movements [26,32,33]. In this study, MD and RD Z-scores were overall lower because LIS patients were scanned in the acute phase of the disease. Nevertheless, some patients showed higher MD and RD Z-scores than controls for a few white matter fiber tracts. This might reflect the reduction of intracellular oedema in these areas, the loss of surrounding microarchitecture and the demyelination process.

Within the whole white matter fiber tracts, DT-MRI statistical analysis highlighted the most injured tracts. The statistical analysis was based on several rather strong assumptions and choices. First, we considered the white matter fiber tracts as the statistical units and the subjects were considered as repeated data across tracts, while the reverse would have been a more natural model. This choice was based on the fact that the number of subjects would inevitably be low compared to the number of tracts and that nevertheless, no data would be lost when analysed in this way. Notwithstanding this decision, the small number of subjects may have constituted a limit to the validity of the study, but the results were essentially the same if we included the last five, six or seven patients in the analysis. The mixture model was chosen because we wished to find a pattern of lesions specific to LIS. Our goal was not to find which lesion would predict the presence of LIS or a given clinical anomaly. The use of a Bayesian method made it possible to run a sensitivity analysis on several parameters of the analysis and, of note, we observed that the results were robust to modification of the prior distribution on variances, which indicates that the data are highly informative compared to the prior information specified in the model. A more classical cluster analysis here would probably have been less informative since it would not have yielded the tract’s probability of being in a specific abnormal pattern or group of abnormal tracts.

The most injured white matter fiber tracts highlighted were the medial lemniscal tracts and the superior cerebellar peduncles, and to a lesser extent the corticospinal tracts.

In agreement with DT-MRI statistical analysis, the anatomical analysis detected two of these three pathways as injured for the majority of LIS patients: the medial lemniscus and the corticospinal tracts, which were logically more injured than the other because they crossed the lesion responsible for LIS in the ventral part of the pons. Anatomical analysis detected superior cerebellar peduncle injuries for only one patient, probably related to a disconnection mechanism and not to an anatomical lesion responsible for the LIS. Therefore, anatomical analysis detected infratentorial lesions, in agreement with DT-MRI statistical analysis results, and seemed to detect more infratentorial white matter fiber tracts injured than DT-MRI analyses (Table 2). Indeed, DT-MRI analyses were limited to the study of 48 white matter fiber tracts provided by the JHU DTI-based white-matter atlases, as we had defined at the beginning of the study in order to have reproducible data between the individuals. This is why the majority of injured white matter fiber tracts detected by anatomical analysis were in fact not studied by DT-MRI analysis. However, the morphological study revealed no supratentorial lesions, except for patient 6 who had few supratentorial abnormalities, while DT-MRI metrics indicated diffuse supratentorial white matter changes. This suggests that DT-MRI and subsequent quantitative tract-based statistics provide a more sensitive approach for depicting microstructural white matter alterations in LIS patients as compared to conventional morphological imaging. This is in line with observations from previous brain imaging studies of other neurological pathologies [22].

Our DT-MRI analysis reveals global supratentorial white matter fiber tract abnormalities in LIS patients. This discovery could open new prospects for pathophysiological mechanisms in LIS patients’ symptoms. Few neuropsychological studies have assessed cognitive function in locked-in syndrome survivors. The first investigations [35–37] emphasised the preservation of cognitive functions. Conversely, recent studies have highlighted specific cognitive impairments in LIS, such as impaired attention, low-speed processing, impairment of perceptual organisation skills, mild inefficiency in new learning, a moderate reduction in executive functions [8,9], motor imagery defects, impaired recognition of negative facial expressions, pathologic laughter and crying, hallucinations and delusions [10–13,38]. Cognitive dysfunctions in LIS are a recent discovery, hence the pathophysiological mechanism has not been fully elucidated. It was surprising to find cognitive disorders among patients suffering from infratentorial lesions, since mechanisms leading to cognitive alterations usually involved supratentorial lesions. To explain this, some authors hypothesise that damage to corticopontocerebellar pathways could involve cognitive impairment [10,13,39]. Corticopontocerebellar pathways, which include corticopontine and pontocerebellar pathways, arise from associative areas in the dorsolateral and dorsomedial prefrontal cortex, posterior parietal region, superior temporal, posterior parahippocampal and from the cingulate gyrus. Therefore, pontocerebellar pathways relay information from the pons to the cerebellum. In return, there is a cerebellar feedback loop through the thalamus to the cerebral cortex including the cerebellothalamic and thalamocortical systems, which are directed to the same associative areas from which they originate. Conson et al. hypothesise that the functional interruption of parietocerebellar connections could account for motor imagery defects in LIS patients [10]. Corticopontocerebellar pathways disconnection could also impair recognition of negative facial expressions and be involved in LIS patients pathologic laughter and crying [12,13]. In 1998, Schmahmann and Sherman reported cerebellar cognitive affective syndrome in 20 patients with diseases within the cerebellum. This clinical entity includes disturbances of executive functions, impaired spatial cognition, personality changes, inappropriate behaviour and language deficits [39]. Adults with acute focal cerebellar lesions or isolated brainstem stroke were reported to suffer from impairment in executive and attentional functions, which evolved to become subtle and disappear in the late phase [40–43]. Thus, cognitive disturbances caused by cerebellar injuries may involve the cerebrocerebellar circuit, which includes the corticopontine and pontocerebellar pathways.

A new prospect could be that some white matter fiber tract injuries highlighted in this early MRI workout study could account for the cognitive disturbances previously described in LIS patients, by injuring some corticopontocerebellar pathways. Very recent studies point in this direction, showing some selective supratentorial cortical volume loss in LIS patients [38,44], especially those with hallucination and delusions. This reinforces the idea that a lesion of the ventral pons, interrupting corticopontocerebellar pathways, could lead to cognitive deficits. A limitation of this hypothesis in the present study is the lack of cognitive evaluation in LIS patients’ studies. Future studies are needed to identify both cognitive dysfunctions and white matter fiber tract injuries in LIS patients and to determinate correlations between them.

## Conclusion

Diffusion tensor magnetic resonance imaging highlighted diffuse supra- and infratentorial white matter fiber tract injuries in consecutive early locked-in syndrome patients. The most injured pathways–the medial lemniscal tracts, corticospinal tracts and superior cerebellar peduncles–cross the ventral part of the pons, the anatomical region responsible for LIS. This finding might contribute to our understanding of cognitive dysfunction in LIS, although the specific pathophysiological mechanisms still remain unknow.

## Supporting information

S1 File FA Z-scores of the 48 white matter fiber tracts studied with DT-MRI for LIS patients 1, 2, 3, 4, 5, 6 and 7. Abbreviations: R, right; L, left; X, indicates that Z-scores cannot been calculated due to noise.
(XLSX)

S2 File MD Z-scores of the 48 white matter fiber tracts studied with DT-MRI for LIS patients 1, 2, 3, 4, 5, 6 and 7. Abbreviations: R, right; L, left; X, indicates that Z-scores cannot been calculated due to noise.
(XLSX)

S3 File AD Z-scores of the 48 white matter fiber tracts studied with DT-MRI for LIS patients 1, 2, 3, 4, 5, 6 and 7. Abbreviations: R, right; L, left; X, indicates that Z-scores cannot been calculated due to noise.
(XLSX)

S4 File RD Z-scores of the 48 white matter fiber tracts studied with DT-MRI for LIS patients 1, 2, 3, 4, 5, 6 and 7. Abbreviations: R, right; L, left; X, indicates that Z-scores cannot been calculated due to noise.
(XLSX)

S1 Table Probability for the 48 white matter fiber tracts injured detected by DTI MRI analyses of belonging to group 2 and their group membership. Group 2 represents the most injured white matter fiber tracts and group 1 represents the least injured white matter fiber tracts. Group 1 or 2 membership: White matter fiber tracts are classified as belonging to group 2 when the probability is greater than 50% and belonging to group 1 otherwise.
(DOCX)

S1 Fig Sagittal, coronal and axial T1-weighted MRI images of LIS patient 1. Arrows indicate the lesion in the brainstem.
(TIFF)

S2 Fig Sagittal, coronal and axial T1-weighted MRI images of LIS patient 2. Arrows indicate the lesion in the brainstem.
(TIFF)

S3 Fig Sagittal, coronal and axial T1-weighted MRI images of LIS patient 3. Red arrows indicate the lesion in the brainstem. Blue arrows indicate the lesion in the cerebellum.
(TIFF)

S4 Fig Sagittal, coronal and axial T1-weighted MRI images of LIS patient 4. Arrows indicate the lesion in the brainstem.
(TIFF)

S5 Fig Sagittal, coronal and axial T1-weighted MRI images of LIS patient 5. Arrows indicate the lesion in the brainstem.
(TIFF)

S6 Fig Sagittal, coronal and axial T1-weighted MRI images of LIS patient 6. Arrows indicate the lesion in the brainstem.
(TIFF)

S7 Fig Sagittal, coronal and axial T1-weighted MRI images of LIS patient 7. Arrows indicate the lesion in the brainstem.
(TIFF)
