# Supplementary material for: Diffusion tensor imaging reveals diffuse white matter injuries in locked-in syndrome patients
Source: PLoS One. 2019 Apr 10;14(4):e0213528. doi: 10.1371/journal.pone.0213528 (PMC6457498; doi:10.1371/journal.pone.0213528)
Supplement: S1 Table — Group 2 represents the most injured white matter fiber tracts and group 1 represents the least injured white matter fiber tracts. Group 1 or 2 membership: White matter fiber tracts are classified as belonging to group 2 when the probability is greater than 50% and belonging to group 1 otherwise. (DOCX) [file pone.0213528.s005.docx]

**S1 Table: Probability for the 48 white matter fiber tracts injured detected by DTI MRI analyses of belonging to group 2 and their group membership.**

| **Injured white matter fiber tracts detected by**  **DTI MRI** | **FA** | **MD** | **Probability of belonging to group 2 (%)** | **Group 1 or 2 membership** |
| --- | --- | --- | --- | --- |
| **Anterior corona radiata left** | -3.82 | -4.69 | 5.28 | 1 |
| **Anterior corona radiate right** | -3.68 | -4.69 | 4.84 | 1 |
| **Anterior limb of internal capsule left** | -3.61 | -3.59 | 3.98 | 1 |
| **Anterior limb of internal capsule right** | -3.76 | -3.54 | 4.36 | 1 |
| **Body of corpus callosum** | -3.84 | -3.10 | 4.16 | 1 |
| **Cerebral peduncle left** | -4.39 | -2.97 | 5.20 | 1 |
| **Cerebral peduncle right** | -4.69 | -2.77 | 4.60 | 1 |
| **Cingulum cingulate gyrus left** | -3.57 | -4.55 | 5.46 | 1 |
| **Cingulum cingulate gyrus right** | -3.66 | -4.55 | 5.10 | 1 |
| **Cingulum hippocampus left** | -2.87 | -4.52 | 4.34 | 1 |
| **Cingulum hippocampus right** | -2.76 | -5.05 | 4.50 | 1 |
| **Corticospinal tract left** | -5.40 | -4.41 | 20.62 | 1 |
| **Corticospinal tract right** | -6.26 | -5.22 | 46.50 | 1 |
| **External capsule left** | -3.49 | -4.29 | 4.80 | 1 |
| **External capsule right** | -3.73 | -4.27 | 5.16 | 1 |
| **Fornix column and body** | -3.68 | -3.03 | 3.36 | 1 |
| **Fornix Stria terminalis left** | -4.32 | -3.85 | 5.26 | 1 |
| **Fornix Stria terminalis right** | -4.24 | -3.98 | 6.04 | 1 |
| **Genu of corpus callosum** | -4.08 | -2.61 | 4.96 | 1 |
| **Inferior cerebellar peduncle left** | -4.23 | -4.37 | 6.66 | 1 |
| **Inferior cerebellar peduncle right** | -4.01 | -5.01 | 6.96 | 1 |
| **Medial lemniscus left** | -9.42 | -7.13 | 98.42 | 2 |
| **Medial lemniscus right** | -8.58 | -6.77 | 97.94 | 2 |
| **Middle cerebellar peduncle** | -4.24 | -4.13 | 6.08 | 1 |
| **Pontine crossing tract** | -4.49 | -4.96 | 9.30 | 1 |
| **Posterior corona radiata left** | -4.11 | -5.11 | 7.84 | 1 |
| **Posterior corona radiate right** | -4.15 | -4.75 | 6.76 | 1 |
| **Posterior limb of internal capsule left** | -4.77 | -2.84 | 5.88 | 1 |
| **Posterior limb of internal capsule right** | -4.38 | -3.10 | 4.40 | 1 |
| **Posterior thalamic radiation left** | -4.39 | -4.11 | 5.58 | 1 |
| **Posterior thalamic radiation right** | -4.03 | -4.49 | 5.16 | 1 |
| **Retrolenticular part of internal capsule left** | -4.14 | -3.87 | 5.30 | 1 |
| **Retrolenticular part of internal capsule right** | -4.19 | -3.97 | 4.82 | 1 |
| **Sagittal stratum left** | -3.97 | -4.29 | 5.60 | 1 |
| **Sagittal stratum right** | -3.81 | -4.23 | 4.74 | 1 |
| **Splenium of corpus callosum** | -4.50 | -2.75 | 4.80 | 1 |
| **Superior cerebellar peduncle left** | -7.44 | -5.31 | 69.32 | 2 |
| **Superior cerebellar peduncle right** | -6.48 | -4.61 | 45.72 | 1 |
| **Superior corona radiata left** | -4.32 | -4.65 | 6.70 | 1 |
| **Superior corona radiate right** | -4.24 | -4.87 | 6.74 | 1 |
| **Superior fronto occipital fasciculus left** | -5.39 | -5.37 | 26.54 | 1 |
| **Superior fronto occipital fasciculus right** | -7.73 | -9.11 | 99.48 | 2 |
| **Superior longitudinal fasciculus left** | -3.68 | -4.64 | 5.24 | 1 |
| **Superior longitudinal fasciculus right** | -3.59 | -4.59 | 4.88 | 1 |
| **Tapetum left** | -3.64 | -2.68 | 3.72 | 1 |
| **Tapetum right** | -3.82 | -2.75 | 3.32 | 1 |
| **Uncinate fasciculus left** | -3.21 | -4.86 | 5.32 | 1 |
| **Uncinate fasciculus right** | -3.37 | -4.22 | 4.26 | 1 |

Group 2 represents the most injured white matter fiber tracts and group 1 represents the least injured white matter fiber tracts.

Group 1 or 2 membership: White matter fiber tracts belong to group 2 when the probability of belonging to group 2 is greater than 50% and belong to group 1 when the probability of belonging to group 2 is less than 50%.
