# Supplementary figures and images for: Diffusion tensor imaging reveals diffuse white matter injuries in locked-in syndrome patients
Source: PLoS One. 2019 Apr 10;14(4):e0213528. doi: 10.1371/journal.pone.0213528 (PMC6457498; doi:10.1371/journal.pone.0213528)

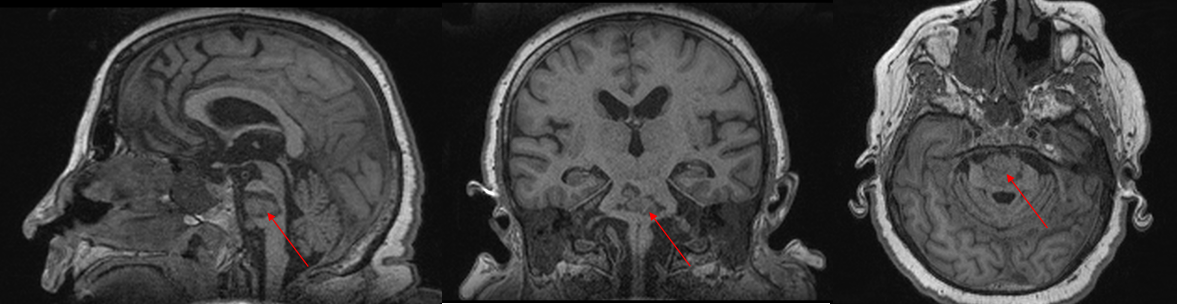

Supplement: S1 Fig — Arrows indicate the lesion in the brainstem. (TIFF) [file pone.0213528.s006.tiff]

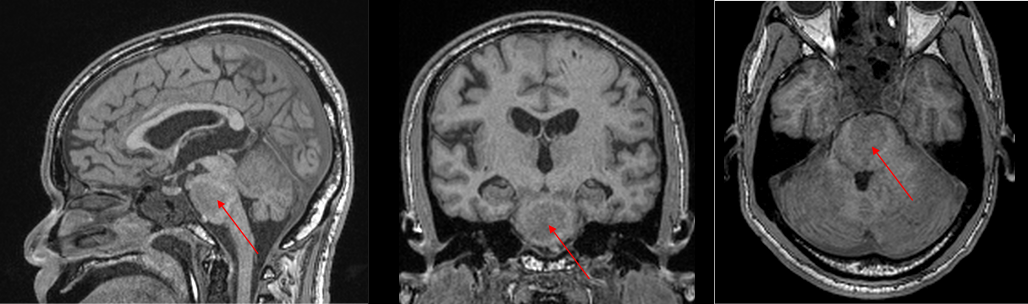

Supplement: S2 Fig — Arrows indicate the lesion in the brainstem. (TIFF) [file pone.0213528.s007.tiff]

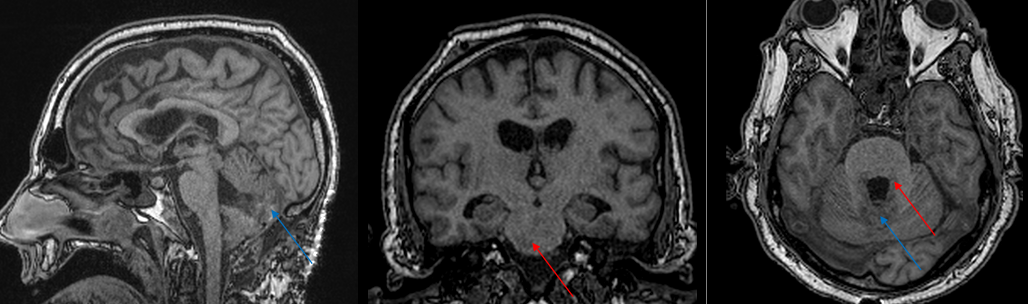

Supplement: S3 Fig — Red arrows indicate the lesion in the brainstem. Blue arrows indicate the lesion in the cerebellum. (TIFF) [file pone.0213528.s008.tiff]

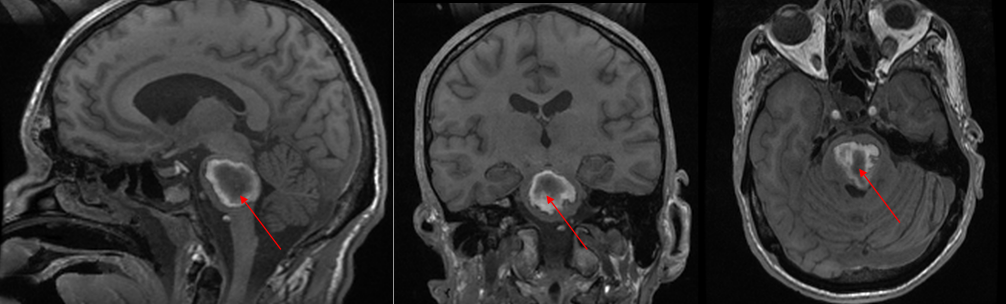

Supplement: S4 Fig — Arrows indicate the lesion in the brainstem. (TIFF) [file pone.0213528.s009.tiff]

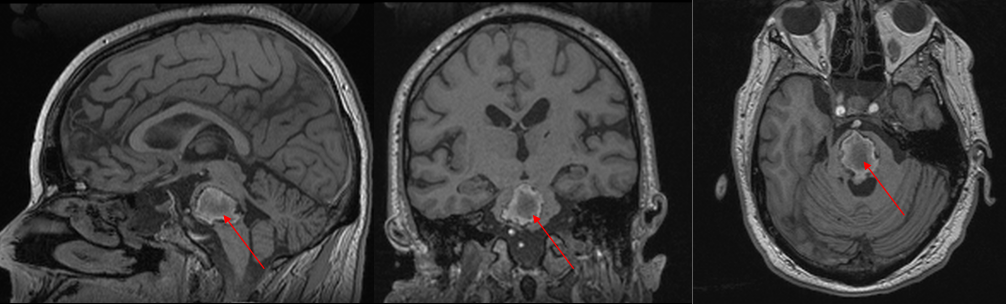

Supplement: S5 Fig — Arrows indicate the lesion in the brainstem. (TIFF) [file pone.0213528.s010.tiff]

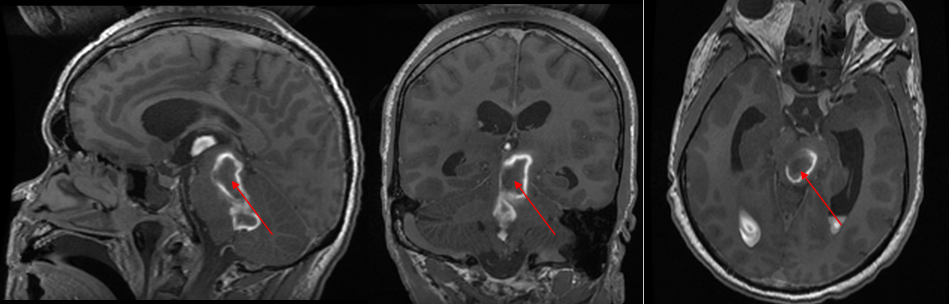

Supplement: S6 Fig — Arrows indicate the lesion in the brainstem. (TIFF) [file pone.0213528.s011.tiff]

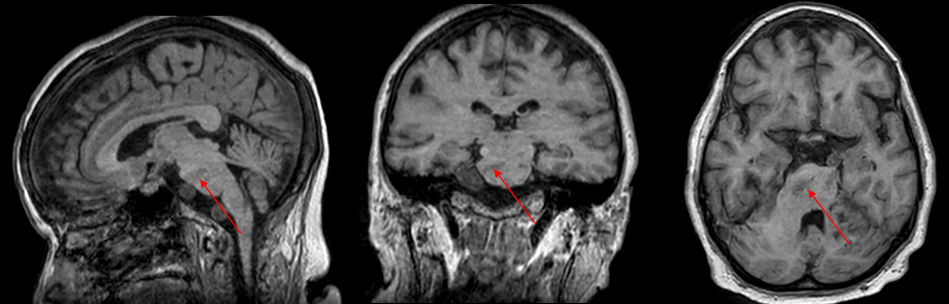

Supplement: S7 Fig — Arrows indicate the lesion in the brainstem. (TIFF) [file pone.0213528.s012.tiff]
